# Precision medicine gets an edge

**DOI:** 10.1016/j.ebiom.2019.12.001

**Published:** 2019-12-16

On Nov 5, 2019, the former UK Health Secretary, Matt Hancock, announced ambitious plans for a whole genome sequencing test that will be made available to 20 000 newborns through the National Health Service (NHS) from early 2020. The pilot study will be undertaken by Genomics England in partnership with the NHS and, depending on its success, this genome sequencing service will be offered to parents at a larger scale. The primary purpose of this testing is to increase the number of rare conditions covered under the NHS newborn bloodspot screening programmes, thus enabling early diagnosis of fatal conditions for which an effective therapeutic option can be actioned in a timely manner to optimise patient care. Genomic data can be used as a reference throughout an individual's lifetime to support diagnoses and guide effective therapy for a variety of diseases, including cancer and other chronic conditions. This important step marks the NHS’ strategy to incorporate highly complex genomic data into routine clinical care, in which disease treatment and prevention will consider an individual's genetic, environmental, and lifestyle needs and their high-level interactions to inform patient-specific health care decisions.

The concept of precision medicine has been on national health care agendas around the world since completion of the Human Genome Project in 2003. The prospect of applying this level of personalised health care at a population-wide level has only started gathering pace in the past decade because of the technological developments that have enabled generation of high-throughput so-called omics datasets and the advent of computational tools such as artificial intelligence and machine learning for effective analysis of the large-scale data. Moreover, the widespread use of wearable, smart technologies allows to capture and interpret longitudinal data on lifestyle and environmental factors and to detect early indicators for health deteriorations and onset of disease. According to a review published as part of a Series on genomic medicine on Aug 5, 2019, in *The Lancet*, genomic sequencing data are increasingly being used to make clinical decisions, and their availability is set to reach over 60 million patients worldwide, over the next 5 years. This *Lancet* Series consists of five timely reviews and explored the efforts, foreseeable limitations, and necessary nationwide frameworks for accelerating implementation of genomic medicine in clinical care.

A hurdle to widespread implementation of precision medicine is the insufficient evidence from clinical trials of the delivery of personalised medicine based on genomic data. In a trial published in May, 2019, in *Nature Medicine,* a team led by Michael Snyder from Stanford University (CA, USA) studied the potential of in-depth longitudinal profiling to uncover a methodology for delivering precision medicine. In this study, multi-omics profiling and clinical assessments of 109 participants susceptible to type 2 diabetes were made for up to 8 years. Physical activity and cardiovascular capacity were assessed and data from physiological testing, wearable monitoring, and completed surveys were collected for all participants. The authors identified more than 67 actionable health factors and exposed key genetic drivers of cardiovascular, metabolic, and oncological risks. Moreover, the rich omics dataset obtained facilitated the development of a predictive framework for insulin resistance and affected the health behaviour of most participants’, leading to a large proportion of them taking up an exercise and diet plan. This intervention shows that combining longitudinal multi-omics profiling with coordinated clinical monitoring is feasible and can lead to patient-tailored health provisions.

An innovative new trial design known as an n-of-1 is gaining interest among researchers who aim to evaluate the varying therapeutic effectiveness and responses in an individual or among group of individuals. In a study published on Oct 9, 2019, in the *New England Journal of Medicine,* researchers from Boston Children's Hospital (MA, USA) reported successful usage of patient-tailored oligonucleotide therapy for treatment of a rare genetic disease in a single patient, within 1 year of initial patient contact. Molecular diagnosis allowed the researchers to identify a novel compound heterozygous mutation (SINE–VNTR–Alu insertion and [1102G→C]) in the patient, leading to a fatal form of neuronal ceroid lipofuscinosis 7 Batten's disease. Using an existing US FDA approved drug (nusinersen), the authors designed a custom antisense oligonucleotide drug, milasen, to amend the missplicing and expression of *MFSD8* in the patient. Rigorous in-vitro testing using the patient's own fibroblasts and in-vivo study in a rat model provided preclinical evidence that the treatment with milasen could substantially modify the disease phenotype by restoring genetic expression and functionality of *MFSD8*. Subsequent application of milasen in the patient led the count and duration of seizures to more than halve and the length of collective seizures time to reduce by more than 80%, with no reported adverse effects. In this case, the clinical and research teams were able to work rapidly to establish an effective treatment option that was tailored to the patient's specific mutation. However, because of existing infrastructure and systemic restrictions—including ethical burden, cost, and manufacturing capacity—this approach is, for now, only scalable to a small group of patients.

In addition, appropriate preclinical disease models designed to provide a deeper understanding of therapeutic responses, allow testing of drug efficacy and adverse effects, and show human relevance will accelerate the implementation of genomic medicine to clinical utility. A research group led by Hans Clevers from Hubrecht Institute (Utrecht, Netherlands) described culturing of patient-derived tubuloids in their March 4, 2019, publication in *Nature Biotechnology*. The authors established a long-term tubuloid culture system derived from human and mouse kidney tissue or human urine. The team modelled BK viral kidney infection, Wilms tumour malignancy, and cystic fibrosis for a proof of concept to show that this patient-derived tumouroid culture closely resembles the primary culture of renal epithelial cells with proximal tubule and can be utilised as various health and diseased human kidney tissue.

A great example of a precision-medicine approach already being employed to deliver a patient-tailored treatment is the use of chimeric antigen receptor (CAR) T-cell therapy for B-cell leukaemia and lymphoma patients. The US Food and Drug Administration has approved two such immunotherapies and a third therapeutic (lisocabtagene maraleucel) is undergoing clinical trials and preliminary indications show promising results. As we noted in our editorial in January, 2019, ongoing efforts being made in the development of natural-killer CAR therapy.

Although a precision-medicine approach is already being implemented in different stages of clinical decision making in some specialities, the policy and regulations that govern the ethics and privacy need to be amended and updated to protect patient privacy and improve guidance on data deposition, distribution, and dissemination. These updates will, in turn, facilitate the development of strategies to improve population health and management of patient care. A broader implementation of precision medicine into routine clinical care could enable some diseases to be tackled at an earlier stage (even before they manifest, in some cases) and perhaps even cured. In this endeavour, *EBioMedicine* will continue to serve as a platform in facilitating the translation of complex omics findings into clinical utility.

*EBioMedicine*

